# Low genetic but high morphological variation over more than 1000 km coastline refutes omnipresence of cryptic diversity in marine nematodes

**DOI:** 10.1186/s12862-017-0908-0

**Published:** 2017-03-07

**Authors:** Daniel Apolônio Silva de Oliveira, Wilfrida Decraemer, Tom Moens, Giovanni Amadeu Paiva dos Santos, Sofie Derycke

**Affiliations:** 1 Faculty of Science, Department of Biology, Ghent University, K.L. Ledeganckstraat 35, 9000 Ghent, Belgium; 2 Royal Belgian Institute of Natural Sciences, OD Taxonomy and Phylogeny, Vautierstraat 29, 1000 Brussels, Belgium; 3 Faculty of Science, Department of Biology, Marine Biology, Ghent University, Krijgslaan 281 (S8), 9000 Ghent, Belgium; 4 Department of Biology, Federal University of Pernambuco, Av. Professor Morais Rego, 1235 - Cidade Universitária, CEP 50670-901, Recife Brazil

**Keywords:** COI, Connectivity, Morphometry, Population genetics

## Abstract

**Background:**

The resilience of ecosystems to negative impacts is generally higher when high gene flow, species diversity and genetic diversity are present. Population genetic studies are suitable to investigate genetic diversity and estimate gene flow between populations. Seaweed beds form a dynamic shallow water ecosystem influenced by climate change and human exploitation, as such, seaweed beds are a particularly powerful model to investigate ecosystem resilience in coastal areas. We studied the population genetic structure of the new nematode species *Paracanthonchus gynodiporata* associated with seaweeds in northeastern Brazil. Nematodes are generally believed to have a limited dispersal capacity because of the lack of planktonic larvae. Yet, they can drift on seaweeds, and water currents might be a natural barrier for their dispersal. Populations of *P. gynodiporata* were sampled over more than 1000 km coastline in regions across major oceanic currents with and without historical exploitation of seaweed.

**Results:**

*P. gynodiporata* is described in an integrative way using mitochondrial and nuclear sequences and morphological data. The 3D model of the head region shows for the first time a detailed view of the ventrosublateral teeth, a character often overlooked in older taxonomic studies of the genus. A total of 17 mitochondrial COI haplotypes were found with one haplotype representing 63 to 83% of the frequencies in each population. AMOVA showed overall little population genetic structure (*F*_*ST*_ = 0.05204), and no genetic subdivision between the populations under the influence of the two different water currents were found. Effects of historical seaweed exploitation on population genetic diversity were not detected. In contrast, significant differences between populations were found in morphometric characters. This discrepancy in genetic and morphological differentiation between populations across 1000 km of coastline is surprising in view of the frequently observed presence of several cryptic species at small geographical scale in other macroalgal associated nematodes.

**Conclusions:**

Our results show that cryptic species are not omnipresent in marine nematode species, suggesting that nematodes associated with seaweeds have been able to disperse over large distances across well-known biogeographic barriers.

## Background

Morphologically similar but genetically distinct species, i.e. cryptic species, are prevalent in many taxonomic groups [1] and have been reported from marine environments since decades [2]. Cryptic species are invoked when genetic variation within species exceeds that typically found between morphologically well-known species. Genetic differentiation between populations within a species depends on selection, genetic drift and gene flow [3]. Widely distributed species can show different patterns of genetic structuring, which are influenced by geographic distance, history and/or natural selection [4].

Many marine organisms were initially thought to have high dispersal capacity because of the passive dispersal potential via currents and the perceived ‘homogeneity’ of marine habitats over extensive spatial scales [5]. However, population genetic structuring among marine populations can be surprisingly high [6, 7], even in organisms with a planktonic larval stage [8]. Organisms which lack planktonic larvae, such as free-living marine nematodes, have a population structuring which strongly varies depending on the species, distance and the environmental conditions [9]. Dispersal can be substantial on fairly small geographical scales (≤100 km), leading to rapid colonization and moderate to little population-genetic structuring [10, 11]. Nematodes that occur on seaweeds can use the seaweed drifting mechanism for their dispersal [12], and this may even occur over oceanic scales [13]. Yet, such long-distance dispersal in marine nematodes is thought to be rare, and substantial cryptic diversity has been observed in marine nematodes associated with macroalgae [10, 14, 15].

Dispersal of marine organisms can be hampered by biogeographic barriers which may result in genetic breaks within species [7]. Well known examples are the Gulf of Mexico and the Atlantic coast in Florida (for instance for the black sea bass [16]), the Indo-Pacific barrier (for instance for populations of the fish *Lutjanus fulvus* [17]), and Point Conception in California (for instance for shark [18]). Along the northern Brazilian coast, known biogeographical barriers are the Amazon-Orinoco Plume and the North Brazilian current which prevent some Caribbean species from dispersing to Brazil [19, 20]. In addition, the split of the South Equatorial current (SEC) in the northeastern coast of Brazil [21] forms two different currents: the above mentioned north Brazil current towards the north and the Brazil Current towards the south. The importance of the latter current as a barrier for dispersal between populations of marine species associated with seaweeds has yet to be clarified.

Seaweed beds can cover areas of thousands of square kilometers [22] but are often discontinuous along the coastline [23, 24]. Although seaweed beds may be hundreds of kilometers apart, such distances may not represent a strong barrier to dispersal of seaweeds [25] and of associated fauna which can drift/raft along with seaweeds [12, 26]. In addition, harvesting of seaweed beds creates a highly dynamic environment [23] where recolonization, founder effects and genetic bottlenecks can affect allele frequencies of the associated fauna [11], which may lead to reduced genetic diversity when compared to areas where no exploitation took place.

One of the most abundant and widespread nematode genera on seaweeds along the NE Brazilian coast is *Paracanthonchus* Mikoletzky (1924; Paracanthonchinae, Cyatholaimidae) [27, 28]. The validity of a number of species within this genus has been debated because of the poor representation of structures in the buccal cavity, among others [29]. Here, we describe the new species *Paracanthonchus gynodiporata* sp. n. which has hitherto only been found associated with seaweeds, in an integrative way based on a large number of specimens (38) from four different populations spanning a wide geographical distribution (> 1000 km). Mitochondrial (COI) and nuclear (18S and the D2/D3 fragment of the 28S rDNA) sequences were obtained and morphometric variation across populations was addressed to capture morphological variation. In addition, a 3D reconstruction of the mouth structure, one of the most important diagnostic characters within the genus, was made. Second, genetic structure and diversity of this new species were investigated using mitochondrial COI sequences of nematodes occurring on seaweed beds separated by the north Brazil and Brazil current [21]. In view of the large genetic structure observed in coastal nematodes from the Atlantic at distances >100 km [9], we expected to find distinct genetic breaks among the Brazilian beaches and across the northeastern split of the south equatorial current. Third, we also investigated the effect of seaweed harvesting on population genetic diversity by comparing samples from two locations with and without historical seaweed harvesting. We expected to find lower genetic diversity in the algal beds that had been disturbed by harvesting because the latter will lead to population bottlenecks.

## Methods

### Field sampling and collection of nematodes

**Fig. 1:**
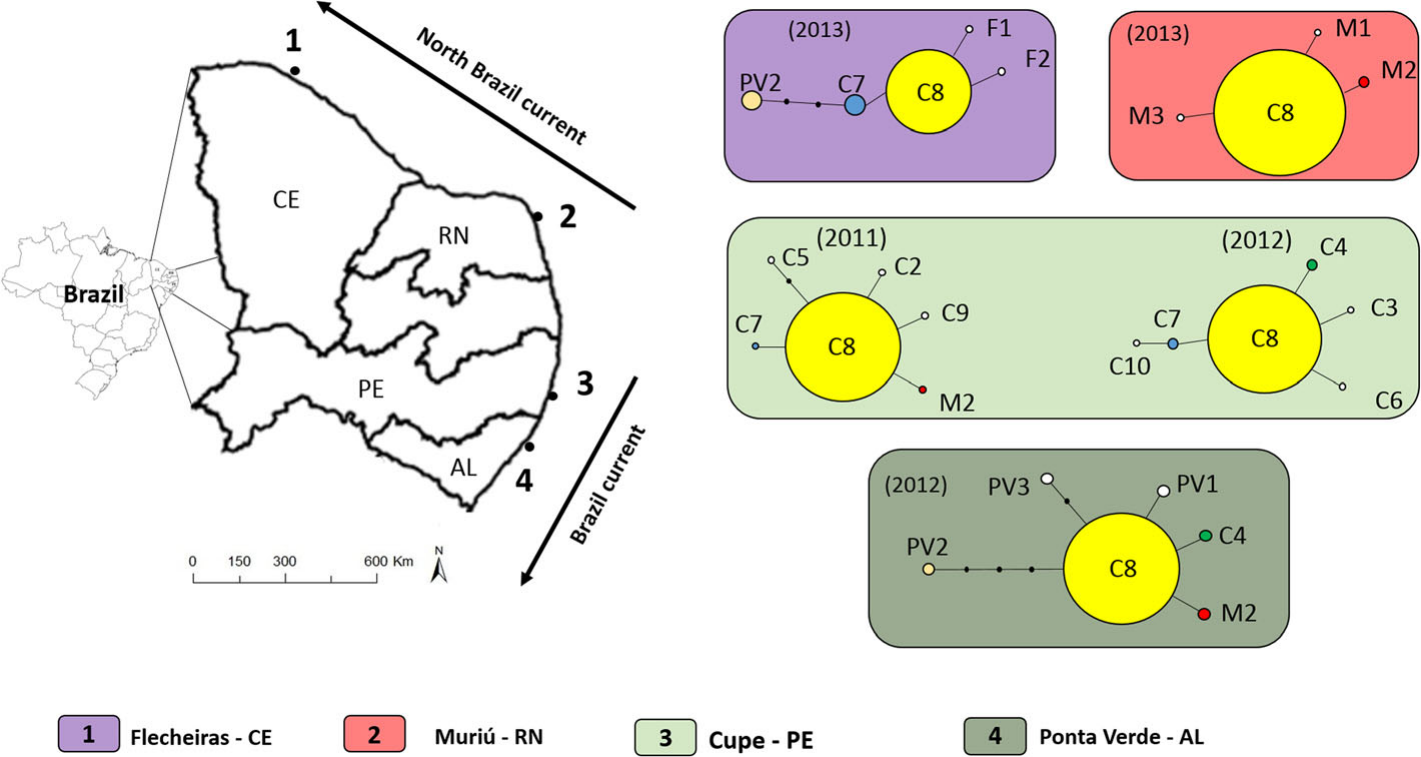
Location of the four sampling sites (indicated by numbers) in the northeastern region of Brazil. The direction of the two main water currents (North Brazil current and the Brazil current) is indicated with arrows. The haplotype networks of *P. gynodiporata* sp. n. in each of the four studied beaches are in boxes with colors corresponding to the sampling sites indicated by the numbers in the map: 1. Flecheiras - Ceará State (CE); 2. Muriú - Rio Grande do Norte State (RN); 3. Cupe - Pernambuco State (PE); 4. Ponta Verde - Alagoas State (AL). Haplotypes are indicated by letters (corresponding to the first letter(s) of the name of the sampling site) followed by numbers (corresponding to the order in which haplotypes were detected in this study) and the size of the circles correspond to the haplotype frequency

Five seaweed samples of the genera *Sargassum* C. Agardh, 1820 and *Gracilaria* Greville, 1830 and five sediment samples were collected from natural seaweed beds on each of four beaches along the northeastern coast of Brazil, spread over a distance of about 1040 km (Fig. 1). All sampling sites were within the Northeastern Brazil ecoregion and sampling took place during the rainy season between April and July. The nematode community associated with both seaweeds is very similar in the northeastern coast of Brazil [28, 30]. The two northernmost beaches are located in Flecheiras (CE) and Muriú (RN), both sampled in 2013, and are under the influence of the north Brazil Current. In those locations, seaweeds from the natural bed, mostly *Gracilaria*, were continuously harvested for about 30 years (historical harvesting), followed by a period of 11 years with no harvesting. Currently, in both locations the natural seaweed beds are no longer harvested. However, in Flecheiras (CE), seaweed cultivation outside the natural seaweed bed started in 2003 in an area smaller than the natural bed, and is still ongoing. The cultivation technique consists of floating ropes of about 25 m long to which the seaweeds are attached. The two southern beaches, Cupe (PE) (sampled in 2011 and 2012) and Ponta Verde (AL) (sampled in 2012), are under the influence of the Brazil current and algal beds are dominated by *Sargassum*. No historical or contemporal harvesting or seaweed cultivation has taken place in these southern locations. Distance between sampled seaweed beds ranged from about 167 to 1045 km (Fig. 1), and each seaweed bed had a total area ranging from ca 0.3 to 4.54 km^2^ (Table 1). Sampling was performed during low tide in the subtidal zone. Only seaweeds attached to the substrate were collected by cutting the base of the holdfast with a knife, put in plastic recipients and fixed with DESS [31]. Five samples containing an entire seaweed plant were collected ca 50 m apart from each other per beach (*Gracilaria*: Flecheiras and Muriú - *Sargassum*: Cupe and P. Verde). Five samples of the top 5 cm of the adjacent bottom sediment were collected using a plastic cylinder with inner diameter of 3.6 cm that was vertically pushed into the sediment. Three seaweed samples and three sediment samples were used for characterization of the nematode community [32], and two were used to collect nematode specimens for the population genetic study. The seaweed samples were washed under a continuous stream of filtered freshwater over a pair of sieves with mesh sizes of 500 and 44 μm.

**Table 1:** Coordinates of the studied locations with the approximate total area of the seaweed bed in km^2^ between brackets, historical background concerning exploitation of the natural seaweed bed and the average relative abundance of *P. gynodiporata* sp. n. in the respective locations. Haplotype occurrence per beach and h (haplotype diversity), π (nucleotide diversity), Tajima’s D and Fu’s Fs neutrality test values with the corresponding *p*-values between brackets of the studied populations at the northeastern Brazilian coast

Beach	Coordinate	Seaweed	Historical background	*P. gynodiporata sp. n. Rel. Abund.*	n° haplotype	Year	*n*	*h*	*π*	*D*	*Fs*
Flecheiras(CE) (1.49 km^2^)	3°13′08″S 39°16′18″W	*Gracilaria*	Exploited	15.56 ± 6.39	5 (F1, F2, C7, C8, PV2)	2013	27	0.5783 ± 0.0961	0.0034 ± 0.0024	−0.3561 (0.3920)	0.0766 (0.5110)
Muriú(RN) (1.86 km^2^)	5°33′43″S 35°14′21″W	*Gracilaria*	Exploited	26.73 ± 8.31	4 (M1, M2, M3, C8)	2013	24	0.3080 ± 0.1180	0.0008 ± 0.0009	−1.4943 (0.0430)	−2.3829 (0.0030)
Cupe(PE) (0.30 km^2^)	8°27′29″S 34°58′58″W	*Sargassum*	Not exploited	37.73 ± 7.16	6 (M2, C2, C5, C7, C8, C9) - (C3, C4, C6, C7, C8, C10)	2011-2012	33 - 25	0.3667 ± 0.122 - 0.3807 ± 0.1058	0.0012 ± 0.0012- 0.0011 ± 0.0011	−2.0875 (0.0040) -1.6480 (0.0160)	−4.3714 (0.0000) -4.0239 (0.0000)
P. Verde(AL) (4.54 km^2^)	9°39′55″S 35°41′54″W	*Sargassum*	Not exploited	22.95 ± 2.47	5 (PV1, PV2, PV3, C4, C8)	2012	20	0.3684 ± 0.1351	0.0019 ± 0.0016	−1.9723 (0.0130)	−1.7287 (0.0590)

*Abbreviation*: *Rel. Abund* relative abundance

### Species selection

The new species *Paracanthonchus gynodiporata* sp. n. was one of the most abundant species on the seaweed samples from the four locations. This new species was systematically absent from all the sediment samples from all four locations in the current study and also in four other locations (Pirambu-CE; Icapuí-CE; São Sebastião-SP; Ubatuba-SC) along the Brazilian coast [32], and was selected for population genetic and phenotypic analyses.

#### DNA extraction, PCR and sequencing of COI, 18S and D2D3 sequences

Because of the very high number of juveniles at different developmental stages and the paucity of adults in the four populations, we were unable to use the same individuals for morphometry and molecular analyses. Instead, individuals of a mix of adults (males and females) and juveniles of *P. gynodiporata* sp. n. from two out of five seaweed samples per beach were taken for molecular processing. No other *Paracanthonchus* species was recorded in our samples. Each individual was stored in 0.5 mL centrifuge tubes with 25 μL Worm Lysis Buffer (50 mM KCl, 10 mM Tris–HCl pH 8.3, 2.5 mM MgCl_2_, 0.45% NP40, 0.45% Tween 20) and stored at -20 °C until DNA extraction. The samples were digested for 1 h at 65 °C and for 10 min at 95 °C with 1 μL of Proteinase K (10 mg mL^−1^). Tubes were centrifuged at maximum speed (21 000 *g*) for 1 min and stored at -20 °C. DNA was subjected to PCR to amplify a 396 bp fragment of the cytochrome oxidase c subunit I (COI) gene with the primers JB3 (5’-TTTTTTGGGCATCCTGAGGTTTAT-3’) and JB5 (5’-GCACCTAAACTTAAAACATAATGAAAATG-3’) [10]. PCR was performed in 25 μL reaction mixtures and contained: 0.125 μL TOPTAQ polymerase (Qiagen), 2.5 μL of 10 X PCR buffer with 15 mM MgCl_2_, 2.5 μL PCR coral load concentrate, 2 μL MgCl_2_ 25 mM, 0.5 μL deoxynucleotide triphosphate (10 mM), 0.125 μL of each primer (25 μM), 1 μL DNA and 16.125 μL sterile distilled water. For COI, the thermocycling conditions were: 94 °C for 5 min; 35 cycles of 94 °C for 30 s, 50 °C for 30 s and 72 °C for 30 s, plus a final extension step at 72 °C for 10 min. The D2D3 region of the large subunit of the nuclear ribosomal DNA was amplified with the primers D2A (5′-ACAAGTACCGTGAGGGAAAGTTG-3′) and D3B (5′-TCCTCGGAAGGAACCAGCTACTA-3′) [15] with amplification starting with a denaturation at 94 °C for 5 min, followed by 40 cycles of denaturation at 94 °C for 30 s, annealing at 58 °C for 30 s and extension at 72 °C for 60 s, and a final extension period of 10 min at 72 °C. The large ribosomal subunit region was amplified using the primers G18S4 (5’-GCTTGTCTCAAAGATTAAGCC-3’) and 4R (5’-GCTTGTCTCAAAGATTAAGCC-3’) with thermocycling conditions of [15]. The sequencing reaction was performed with BigDye Terminator v. 3.1 Mix (PE Applied Biosystems) and under the following conditions: an initial denaturation of 2 min at 98 °C was followed by 40 cycles of denaturation at 98 °C for 10 s, annealing at 50 °C for 5 s, and extension at 60 °C for 60 s. The bidirectional sequences can be found under GenBank accession numbers KX352221 - KX352239. Haplotypes were named after the place where they occurred using the first letter(s) followed by a number which corresponds to the order in which haplotypes were recorded (e.g. Flecheiras (CE) first found haplotype = F1; Ponta Verde-CE second found haplotype = PV2).

### Species description, morphometry and 3D reconstruction of the head region

Digital pictures of eight males and 12 females of *P. gynodiporata* sp. n. from the type location Cupe (PE) mounted in permanent slides were taken at different magnifications using a light microscope (Leica DAS microscope type R) with differential interference contrast (DIC), and equipped with a Leica DFC420 camera. The entire habitus and selected body regions (head, mid-body, and tail) with important taxonomic structures were photographed and measured using Leica Application Suite v. 3.4.1. Slides were deposited in the Royal Belgian Institute of Natural Sciences under the numbers RIT848 (holotype), RIT849 and RIT850 (Paratypes) and in the Zoology Museum at the Faculty of Sciences, Ghent University, under the numbers UGMD 104316. In addition to the type specimens, four, six and four males from Flecheiras (CE), Muriú (RN) and P. Verde (AL), respectively, were measured and 15 somatic and six sexual characters were used to investigate morphometric variability. Within the genus *Paracanthonchus*, there is substantial variation in stoma structure (armature) according to previous descriptions, varying from a single hollow dorsal tooth to one hollow dorsal tooth and two pairs of small ventrosublateral teeth combined or not with three cuticular ridges [29]. The stoma structure with feeding apparatus is an important character to differentiate congeneric species, though not always easy to interpret. To this end, a head section of one male individual of *P. gynodiporata* sp. n. from the type location Cupe (PE) from 2012 was mounted in a glycerol-gelatin mixture (60 g distilled water, 10 g gelatin, 70 g glycerol, and 1.4 g phenol) and observed under a light microscope. In total, 52 pictures at different optical sections of the head were taken. The pictures were used for constructing the 3D model in the software AMIRA 3.1.1. (TGS Software, San Diego, California, USA).

### Data analyses

#### Population genetic structure

The sequence chromatograms from the three markers COI, D2D3, and 18S were investigated with DNASTAR LASERGENE SeqMan v. 7.1.0. Sequences from the three markers were separately aligned by ClustalW. COI sequences were translated to amino acid sequences before the alignment to ensure that no stop codons would be present. P-distances and the number of variable sites were calculated using the software MEGA 6 [33]. Genetic diversity within sampling sites was investigated by calculating nucleotide diversity (*π*) and haplotype diversity (*h*) according to [34–36]. Lower genetic diversity was expected in sampling sites where seaweed was harvested because smaller population sizes lead to increased genetic drift. Population genetic structure was assessed by Analysis of MOlecular VAriance (AMOVA), using the frequencies of the COI haplotypes to calculate overall and pairwise *F*_*ST*_ [37]. The level of population genetic structure followed Wright’s division [38]: little (0.0–0.05), moderate (0.05–0.15), large (0.15–0.25) and very large (above 0.25) genetic differentiation. To investigate whether sequence evolution followed a neutral model, Tajima’s D and Fu’s Fs neutrality tests were performed. When both tests were significantly different from zero, a mismatch analysis was performed by comparing the frequency distribution of the pairwise sequence differences with the expected distribution based on the sudden expansion model to investigate whether the populations experienced an expansion [39]. This is particularly relevant to investigate effects of bottlenecks and population growth in the sampling sites that have been harvested in the past. To investigate whether the North Brazil and Brazil sea currents create a biogeographical barrier for *P. gynodiporata* sp. n., a hierarchical AMOVA was conducted by grouping Flecheiras (CE) and Muriú (RN) in a northern group under the North Brazil current and Cupe (PE) and P. Verde (AL) in a southern group under the Brazil current. The pairwise *F*_*ST*_ *p*-values were corrected based on the sequential Bonferroni method [40]. Additionally, we compared haplotype frequencies from 2011 to 2012 in Cupe (PE) to investigate temporal variation in population genetic structuring. All population genetic analyses were performed using the Arlequin 3.5.1.2 software [36]. To investigate evolutionary relationships and mutational differences between haplotypes, as well as the geographical distribution of haplotypes, a haplotype network was built based on the median joining algorithm implemented in NETWORK 4.6.1.4 [41] and edited with Microsoft PowerPoint software.

#### Phenotypic variability

All morphometric data analyses were performed with the software PRIMER v. 6.1.6 [42]. In all four locations, a high dominance of juveniles and variable but low numbers of females and males were present. Variability in morphometric characters was assessed based on males present in the samples (Flecheiras: 4; Muriú: 6; Cupe: 8; P. Verde: 4). Only from one beach (Cupe) there was a suitable number of both sexes to analyze possible sexual dimorphism (12 females and eight males). The characters used in the analysis were chosen based on what is used in the literature to differentiate congeneric species of the genus *Paracanthonchus* [29] (Table 2). The somatic and sexual characters (copulatory apparatus and precloacal supplements) were analyzed separately (15 somatic and six sexual characters described in Table 2). The dataset was normalized and a dissimilarity matrix based on Euclidean distance was constructed. No transformations were performed. Nonmetric multidimensional scaling (nMDS) and one-way PERMANOVA with fixed factor location were performed. In addition, a similarity of percentages (SIMPER) analysis was performed to detect which characters contributed most to the observed differences, if any, between the different populations. If significant differences were found, the highest ranked characters were compared among populations by performing a one-way ANOVA after verification of the assumptions (Kolmogorov-Smirnov normality test, Levene’s homogeneity test, XY mean and standard variation plot) to test whether the character alone is able to differentiate the populations or whether the differences were a result of a combination of characters. Correlation between morphometric characters was investigated using STATISTICA v. 7 [43].

**Table 2:** Somatic and sexual characters used for the morphometric analysis of *Paracanthonchus gynodiporata* sp. n. from the four studied populations along the northeastern coast of Brazil. Morphometry of the holotype (holo) and paratypes of *Paracanthonchus gynodiporata* sp. n. from Cupe-PE in the northeastern coast of Brazil

	Flecheiras-CE				Muriú-RN				Cupe-PE				P. Verde-AL			
	Min	Max	Average	Std	Min	Max	Average	Std	Min	Max	Average	Std	Min	Max	Average	Std
Somatic																
L	860	952	905	42	779	1090	961	114	800	1119	1059	110	903	1248	1048	160
Pharynx length	123	138	129	7	113	144	126	10	123	152	143	9	127	156	139	13
Ventr. pore dist. ant. end	20.9	29.8	24.3	3.9	21.9	24.0	23.0	0.8	21.0	31.1	27.8	3.1	23.8	28.3	26.5	2.0
Tail length	103	128	114	11	106	132	124	10	114	133	124	6	111	131	120	9
Abd	42.6	52.0	46.5	4.0	35.4	51.0	43.6	5.1	32.2	45.5	41.9	4.2	35.7	44.7	39.3	4.0
Head diameter	21.5	22.6	22.0	0.5	21.4	33.1	27.1	4.0	21.8	25.8	24.0	1.4	21.3	23.0	22.1	0.8
Cephalic Sensilla Length	3.1	3.8	3.5	0.3	3.0	3.3	3.2	0.1	2.8	4.4	3.9	0.5	3.6	5.0	4.2	0.6
Buccal width	7.9	10.4	9.7	1.2	7.1	7.9	7.6	0.3	7.5	10.9	9.2	1.2	7.8	8.7	8.4	0.5
Buccal length	6.8	8.5	7.9	0.8	8.6	11.5	9.4	1.1	7.7	9.7	8.5	0.7	6.1	9.2	7.8	1.3
Amphid. fovea length	8.6	10.2	9.5	0.7	8.2	10.5	9.8	0.9	8.8	10.6	9.5	0.6	9.4	10.1	9.7	0.3
Amphid. fovea width	9.5	11.1	10.5	0.8	9.1	11.8	10.4	0.9	10.0	12.7	11.2	0.8	9.4	11.0	10.2	0.6
Amphid. dist. ant. end	8.6	11.3	10.4	1.2	6.5	8.7	7.9	0.8	8.8	14.2	12.0	1.7	7.8	12.4	9.7	2.1
Pharynx width base	19.6	26.3	24.0	3.1	20.5	26.7	23.2	2.6	18.3	24.6	20.8	1.9	18.3	21.1	19.7	1.3
cbd base pharynx	41.6	47.5	45.3	2.6	40.0	51.5	47.0	4.4	36.0	46.6	42.2	3.0	35.7	41.8	38.9	2.6
Body width	43.5	55.2	50.6	5.0	46.6	60.2	55.5	4.8	40.4	54.2	48.5	4.0	41.4	52.5	45.4	5.1
a	16.5	21.9	18.1	2.6	15.3	19.7	17.3	1.7	19.8	23.4	21.8	1.2	21.8	24.3	23.0	1.2
b	6.2	7.6	7.0	0.6	6.9	8.3	7.6	0.5	6.5	7.8	7.4	0.4	6.7	8.4	7.5	0.8
c	7.5	8.5	8.0	0.4	7.0	8.3	7.7	0.6	7.0	9.5	8.6	0.8	8.1	9.5	8.7	0.7
Sexual																
Spicule length	33.7	34.4	34.0	0.3	35.3	42.8	37.8	3.6	35.6	45.6	40.6	3.5	37.9	41.7	39.3	1.6
Gubernac. length	31.9	33.8	33.1	0.9	32.6	37.0	34.7	1.5	35.0	37.3	36.4	0.6	34.2	38.6	36.3	1.8
Supplement length 4	20.2	23.1	21.2	1.3	18.5	25.7	23.2	2.6	21.7	30.8	27.2	3.0	22.5	27.6	24.4	2.3
Supplement length 3	20.1	22.2	20.8	1.0	17.8	25.2	23.1	2.9	23.2	28.5	26.6	1.6	22.6	28.3	24.7	2.6
Supplement length 2	20.1	20.9	20.5	0.3	14.3	25.0	21.7	3.9	20.2	29.1	25.9	2.7	22.4	26.2	24.3	1.7
Supplement length 1	18.3	20.4	19.5	1.0	14.5	22.8	20.0	2.9	16.8	24.8	23.0	2.6	21.3	24.9	23.1	2.0

*Abbreviations*: *L* body length, *Ventr. pore dist. ant. end.* ventral pore distance from the anterior end, *abd* body diameter at anus level, *Amphid dist. ant. end* Amphid distance from the anterior end, *cbd base pharynx* corresponding body diameter at the base of the pharynx, *a* body length/body width, *b* body length/pharynx length, *c* body length/tail length

## Results

### Population genetic structure

**Fig. 2:**
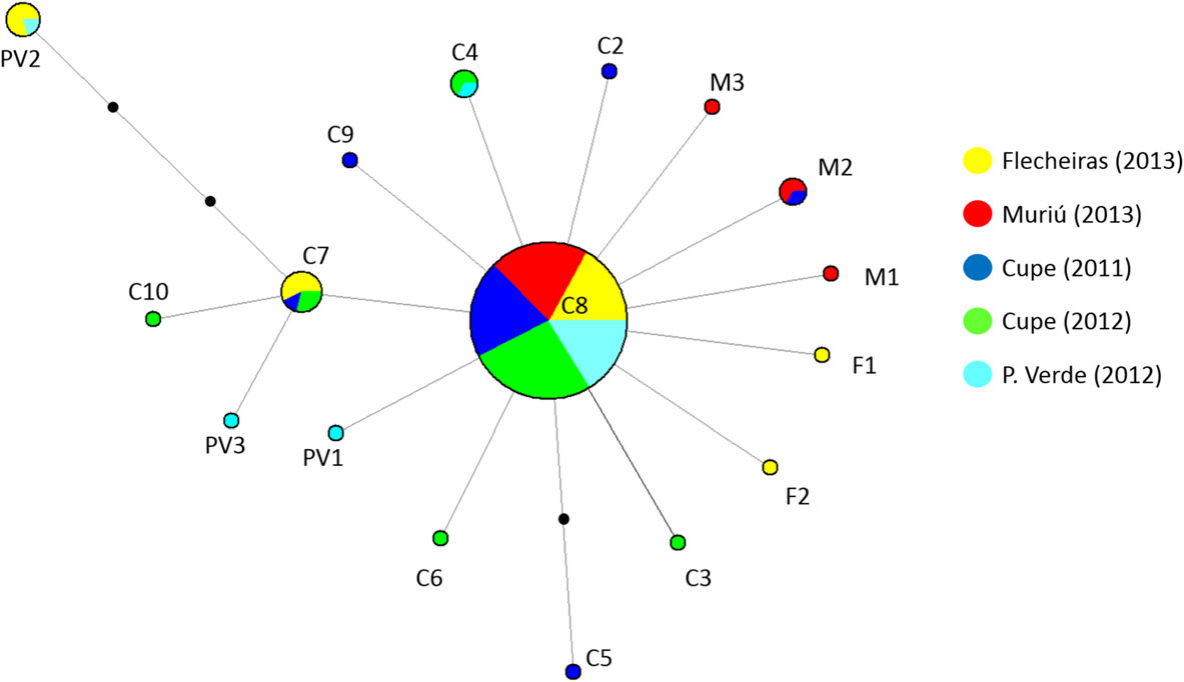
Overall haplotype network of *P. gynodiporata* n. sp from the four studied beaches. The size of the circles correspond to the haplotype frequency in the total dataset. Beaches are represented by different colors

In total, 27 (Flecheiras (CE)), 24 (Muriú (RN)), 25 (Cupe (PE 2011)), 33 (Cupe (PE 2012)) and 20 (P. Verde (AL)) individuals from *P. gynodiporata* n. sp. yielded good COI sequences (396 bp sequences and alignment with 19 variable sites). The best blastn identity for all COI sequences was *Paracanthonchus* sp. (FN998914.1, identity 87–88%, query cover 59%). In total, 17 COI haplotypes were found, with haplotype C8 being the most abundant in all four locations (average 77% ± 8). Only four other haplotypes were shared between at least two beaches, while the remaining 12 haplotypes were restricted to a single location and occurred at low frequencies (Figs. 1 and 2). The p-distances ranged from 0.003 to 0.015 and number of differences ranged from one to six base pairs. The haplotype network further revealed a low number of mutations between haplotypes and a star-shaped pattern with no geographical clustering of haplotypes (Fig. 2). The AMOVA analysis revealed a little but statistically significant genetic structuring (*F*_*ST*_ = 0.05204; *p* = 0.00391). The pairwise analysis showed moderate separation between the populations of Flecheiras (CE) and Muriú (RN), and between Flecheiras (CE) and Cupe (PE) 2012 (Table 3). Within Cupe (PE), no significant temporal variation in haplotype composition was observed between 2011 and 2012 (*F*_*ST*_ = 0.00091; *p* = 0.38739).

**Table 3:** Pairwise *F*_*ST*_ values between the four populations of *P. gynodiporata* sp. n. in Flecheiras (CE), Muriú (RN), Cupe (PE) and P. Verde (AL)

	Flecheiras	Muriú	Cupe 11	Cupe 12	P. Verde
Flecheiras					
Muriú	**0.13125**				
Cupe 11	0.10175	0			
Cupe 12	**0.09177**	0.02486	0.00091		
P. Verde	0.02502	0.020	0	0	

Signifcant *F*_*ST*_ values after Bonferonni correction are indicated in bold. Negative values were converted to zero

**Fig. 3:**
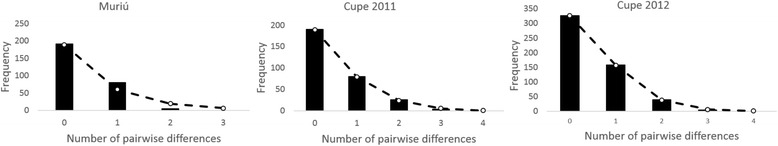
Mismatch distribution of the pairwise differences of the haplotypes occurring in Muriú (RN) 2013, Cupe (PE) 2011 and Cupe - PE 2012. Muriú - RN (SSD = 0.00801, *p* = 0.409; Raggedness = 0.23591, *p* = 0.641) and Cupe - PE, 2011 and 2012 (SSD = 0.00007, *p* = 0.872; Raggedness = 0.17240, *p* = 0.745 - SSD = 0.00003, *p* = 0.95730; Raggedness = 0.15770, *p* = 0.563)

Haplotype networks for each location showed the same pattern as the overall network, with one dominant haplotype and a low number of rare haplotypes with few mutations between them. Genetic diversity appeared to be higher in Flecheiras (CE) Beach (*h* = 0.5783 ± 0.0961), where the seaweed bed was considered to be most impacted because of historical and ongoing seaweed exploitation; however, the standard deviation overlapped with those of the diversity estimates observed in the other locations except Muriú (RN) (Table 1). Tajima’s *D* and Fu’s *Fs* neutrality test statistics were negative and significantly different from zero for the beaches Muriú (RN) and Cupe (PE - 2011 and 2012) (Table 1), and point to recent expansion or purifying selection. The mismatch distribution analyses were unimodal and fitted the sudden expansion model, indicating that those populations experienced a recent sudden expansion (Fig. 3 Muriú – RN: *SSD* = 0.00801, *p* = 0.409, Raggedness = 0.23591, *p* = 0.641; Cupe – PE, 2011 and 2012: *SSD* = 0.00007, *p* = 0.872, Raggedness = 0.17240, *p* = 0.745 and *SSD* = 0.00003, *p* = 0.95730, Raggedness = 0.15770, *p* = 0.563). A significant but little genetic structure was observed (*F*_*ST*_ = 0.05204; *p* = 0.00391 ± 0.00185) between the northern group under the influence of the north Brazil current (Flecheiras (CE)) and Muriú (RN)) and the southern group under the influence of the Brazil current (Cupe (PE - 2011, 2012) and P. Verde (AL)), indicating that the split of the South Equatorial Current along the Brazilian coast imposes only a weak biogeographical barrier for the *P. gynodiporata* sp. n. populations. The D2D3 (747 bp; sequences: 4 in Cupe (PE) and 17 in P. Verde (AL)) and 18S (914 bp; sequences: 8 in Cupe (PE) and 19 in P. Verde (AL)) sequences were identical. The best blastn identity for D2D3 sequences was *Paracanthonchus* sp. (KX270432.1, identity 82%, query cover 100%) while for 18S it was *Paracyatholaimus intermedius* (AJ966495.1, identity 94%, query cover 98%).

### Phenotypic variability

**Fig. 4:**
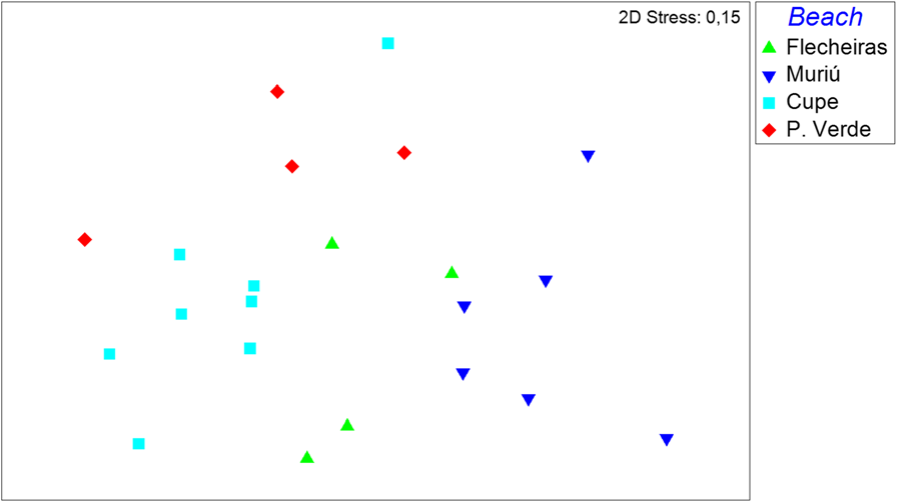
Non-metric multidimensional scaling of the somatic characters among the individuals of *P. gynodiporata* sp n. populations of Flecheiras - Ceará State (CE), Muriú - Rio Grande do Norte State (RN), Cupe - Pernambuco State (PE) and Ponta Verde - Alagoas State (AL) along the Brazilian coast. Used characters: body length, pharynx length, distance of ventral pore to anterior end, tail length, anal body diameter, head diameter, cephalic sensilla length, buccal width, buccal length, amphidial fovea, length, amphidial fovea width, distance of amphidial fovea to anterior end, width at the base of the pharynx, corresponding body diameter at the base of the pharynx, body width, body length divided by the width, body length divided by pharynx length, body length divided by tail length, spicule length, gubernaculum length, length of the 4th, 3rd, 2nd and 1st precloacal supplements

Morphometric variation among males from different populations was significant (PERMANOVA: Pseudo-*F* = 4.7107; *p* < 0.001; Fig. 4). However, significant non-overlapping measurements were restricted to three characters. Buccal cavity width (one-way ANOVA: *F* = 5.657; *p* = 0.006), distance of the amphidial fovea from anterior end (one-way ANOVA: *F* = 8,5366; *p* < 0.001), and cephalic setae length (one-way ANOVA: *F* = 7,048; *p* = 0.002) were significantly different and non-overlapping between Flecheiras (CE) and Muriú (RN); Muriú (RN) and Cupe (PE); and Muriú (RN) and P. Verde (SIMPER and Tukey pairwise comparison). In contrast, specimens between Flecheiras (CE) and P. Verde (AL) and between Cupe (PE) and P. Verde (AL) did not differ significantly. With respect to sexual characters, substantial variability was observed in the precloacal supplement length (PERMANOVA: Pseudo-*F* = 4,4825; *p* = 0.002). The anteriormost precloacal supplement (SP4) was the top ranked character that contributed to the differences between populations: SP4 of individuals from Cupe was longer than that of individuals from Flecheiras (SIMPER: Cupe (PE) x Flecheiras (CE) = 25.65% contribution). In addition, the posterior most precloacal supplement (SP1) of individuals from P. Verde (AL) was longer than the one of individuals from Flecheiras (CE) (SIMPER: P. Verde (AL) x Flecheiras (CE) = 23,03% contribution), and SP3 of the individuals from Cupe (PE) was longer than the one of specimens from Muriú (SIMPER: Cupe (PE) x Muriú (RN) = 30% contribution). Spicule and gubernaculum did not differ significantly among populations. Because of considerable overlap, no single sexual character by itself could distinguish individuals of one location from those of other locations. The character measurements are presented in Table 2.

### Species description

Genus *Paracanthonchus* Bastian, 1865

**Fig. 5:**
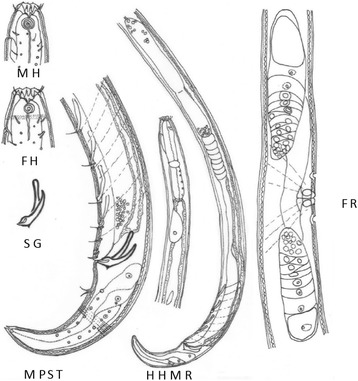
*Paracanthonchus gynodiporata* sp. n. Abbreviations: MH - Head region male holotype; FH - head region female paratype showing sexual dimorphism in amphidial fovea; SG - detail of holotype spicule and gubernaculum; MPST - male holotype posterior region with precloacal supplements and tail; HHMR - holotype habitus with male reproductive system; FR - female paratype reproductive system

**Fig. 6:**
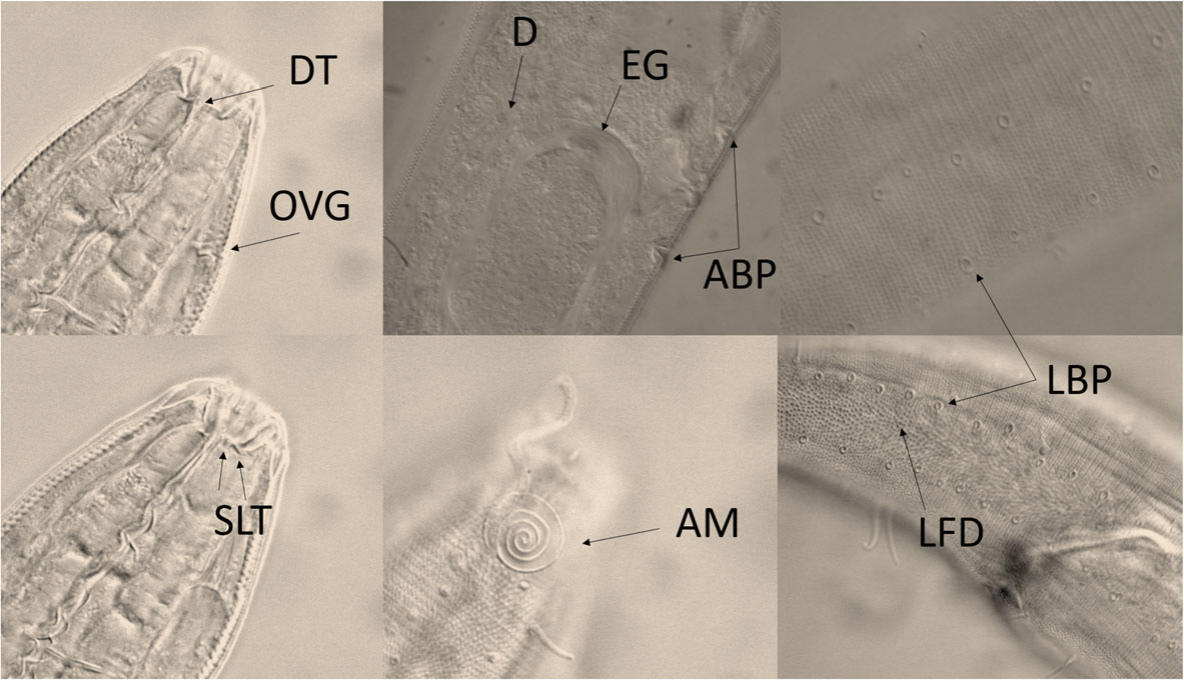
*Paracanthonchus gynodiporata* sp. n. Abbreviations: DT - dorsal hollow tooth; OVG - sclerotized outlet of the ventral gland; SLT - ventrosublateral teeth; D - detail of diatom in the intestine; EG - egg in the uterus; ABP - pre and post-advulval body pores; AM - male amphidial fovea; LBP - longitudinal rows of large body pores bordering the lateral field; LFD - lateral field and differentiation

**Fig. 7:**
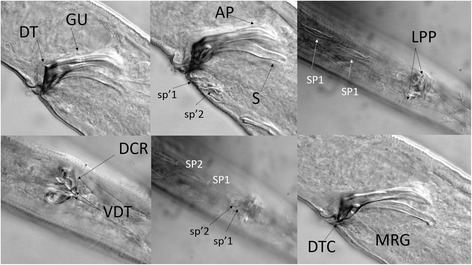
*Paracanthonchus gynodiporata* sp. n. Abbreviations: DT - Gubernaculum with dorsal thorn of crura; GU - gubernaculum; DCR - dorsal crura ridge; VDT - ventral view of the dorsal thorns; AP - gubernaculum apophysis; S - spicule; LPP - lateral pointed protuberance; DTC - gubernaculum showing distal thorn of crura; MRG - mid-rib gubernaculum; SP - precloacal supplements; sp’ minute precloacal supplements

**Fig. 8:**
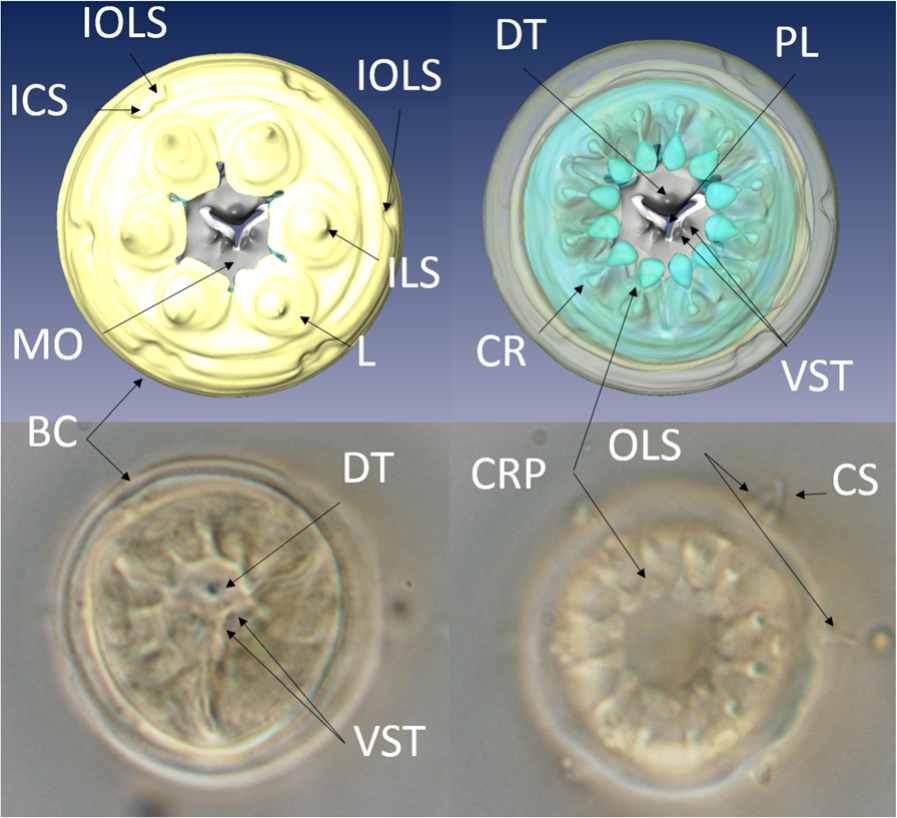
*Paracanthonchus gynodiporata* sp. n. Detailed reconstruction of the head region showing the mouth armature indicating the number and position of teeth, especially the ventrosublateral, which are variable and an important feature in the literature. The numbers between brackets correspond to the number of the referred structure when different from 1. Abbreviations: ICS - Insertion of the cephalic setae (4); IOLS - insertion of the outer labial setae (6) which is at the same level of the outer labial setae composing a single circle with 10 sensilla (4 + 6); ILS - inner labial sensilla (6); MO - mouth opening; L - labium (6); DT - dorsal hollow tooth; VST - ventrosublateral teeth; PL - pharynx lumen; BC - body cuticle; CR - cheilorhabdia (12); CRP - prolongation of the cheilorhabdia beneath labial cuticle (12); OLS - outer labial setae; CS - cephalic setae

*Paracanthonchus gynodiporata* sp. n. (Figs. 5, 6, 7 and 8, Table 4).

**Table 4:** Morphometry of the holotype (holo) and paratypes of *Paracanthonchus gynodiporata* sp. n. from Cupe-PE in the northeastern coast of Brazil

	RIT848				RIT849			RIT850			UGMD 104316		
	Male (Holo)	Female (NG)	J4 stage	Male	Female (NG)	J3 stage	Male	Male	Female (G)	Female (G)	Male	Female (G)	J3 stage
L	1099	1238	796	1072	1001	819	1116	1146	1184	1145	1060	1075	500.83
Distance vulva anterior end	n/a	500	373	n/a	466	n/a	n/a	n/a	521	552	n/a	488	n/a
Pharynx length	151	156	132	144	154	131	147	162	172	172	151	160	97
Ventr. pore. Dist. ant. end	26.0	24.3	n/a	26.6	33.1	27.9	30.3	30.6	29.5	32.7	27	26	28.3
Tail length	130	126	107	120	120	115	126	130	132	132	121	121	76
abd	40	39.1	34.3	38.6	36.2	37.8	41.1	43.4	39	39.9	40.6	37.7	24.9
Head diameter	22.4	22.6	24.9	21.4	22.1	22.9	21.5	22.9	24.5	24.2	20.5	25.9	15.3
Sensila Length	4.5	3.5	3	3.7	4.7	2.5	3.7	4.6	4.9	4.4	2.9	4.2	2.3
Buccal width	9.7	10.8	n/a	10.4	11.7	n/a	9.6	8.9	10.6	11.1	9.8	10.8	6.8
Buccal length	8.5	8.7	n/a	8.9	10	n/a	8.5	9.6	9.9	10.8	9.5	10.3	6.6
Amphid. fovea length	10	7.3	n/a	9.8	6.7	n/a	9.5	9.2	6.9	7.1	9.3	7.4	4.
Amphid. fovea width	10	7.4	6.2	10.5	8.1	n/a	10.7	9.6	8.3	8.6	9.2	7.8	4.8
Amphid. dist. Ant. end	11.5	11.5	n/a	11.2	12	n/a	11.3	11.4	11.5	1188	12.9	12.3	9.1
Pharynx width base	21	24.5	21.9	19.9	25.5	18.6	19.5	23.4	28.7	27.5	18.9	26.9	15.9
cbd base pharynx	40	46.1	40.6	42.0	47.4	38.8	39.9	47.1	52.80	48.6	40.9	46.6	29.5
Pre-vulvar body pore	n/a	16.5	14.8	n/a	16.2	n/a	n/a	n/a	16.5	17.2	n/a	15.6	n/a
Post-vulvar body pore	n/a	15.5	12.6	n/a	15.6	n/a	n/a	n/a	16.8	16.3	n/a	16.1	n/a
Body width	40	55.9	46	46.8	58.5	41.4	48	n/a	65.90	63.6	50.4	58.2	30.8
Spicule length	40	n/a	n/a	38.9	n/a	n/a	39.2	39.5	n/a	n/a	42.1	n/a	n/a
Gubernac. length	34.6	n/a	n/a	36.1	n/a	n/a	35.9	40.8	n/a	n/a	34.5	n/a	n/a
Supplement length 4	21.5	n/a	n/a	21.7	n/a	n/a	23.2	22.5	n/a	n/a	23.7	n/a	n/a
Supplement length 3	25.4	n/a	n/a	24.9	n/a	n/a	25.2	23.2	n/a	n/a	24.3	n/a	n/a
Supplement length 2	24.6	n/a	n/a	25.4	n/a	n/a	25.1	24.4	n/a	n/a	24.8	n/a	n/a
Supplement length 1	26	n/a	n/a	25.8	n/a	n/a	25.7	25.5	n/a	n/a	25.8	n/a	n/a

*Abbreviations*: *L* body length, *Ventr. pore dist. ant. end.* ventral pore distance from the anterior end, *abd* body diameter at anus level, *Amphid dist. ant. end* Amphid distance from the anterior end, *cbd base pharynx* corresponding body diameter at the base of the pharynx, *a* body length/body width, *b* body length/pharynx length, *c* body length/tail length. The codes RIT848, RIT849, RIT850 and UGMD 104316 correspond to one slide each

*Holotype*. Male (Fig. 5).

*Paratypes*. Five males, five females, three juveniles at fourth stage (two molting specimens, one to male, one to female).

Type locality. Brazil, Pernambuco State, Cupe Beach (8°27′29′S 34°58′58″W), subtidal zone, associated with brown seaweed *Sargassum polyceratium.*

*Other Localities*. Praia de Flecheiras, Trairí – Ceará – Brazil (3°13′08″S 39°16′18″W); Praia de Muriú, Ceará-Mirim – Rio Grande do Norte – Brazil (5°33′43″S 35°14′21″W), Praia de Ponta Verde, Maceió – Alagoas – Brazil (9°39′55″S 35°41′54″W).

Sequences from type location:

Type Haplotypes: COI – KX352225 (C2), KX352226 (C3), KX352227 (C4), KX352228 (C5), KX352229 (C6), KX352230 (C7), KX352231 (C8), KX352232 (C9), KX352233 (C10), KX352235 (M2).

Type Genotypes: 18S - KX352221; D2D3 - KX352222

Life Science Identifier (LSID): urn:lsid:zoobank.org:pub:B6FBE6B8-B482-4206-A9D3-842A7014A505.

Etymology: The species name refers to the pre- and post-advulvar body pores in the female (Fig. 6).

The number of sequences available for the genus *Paracanthonchus* is very restricted in GenBank. COI (396 bp): 317 conserved and 76 variable sites compared to *Paracanthonchus* sp. (FN998914.1) 18S (914 bp): 848 conserved and 61 variable sites compared to *P. caecus* (AF047888.1) D2D3 (747 bp): 532 conserved and 127 variable sites compared to *Paracanthonchus* sp. (KJ638031.1).

### Diagnosis and relationships

Body medium-sized (779.3 – 1058.5 μm), largely cylindrical with rounded truncated head with conical tail; body cuticle with transverse rows of fine dots, slightly larger at level of lateral field, more visible posterior to the neck region; amphidial fovea ventrally spiral, smaller in females with 3.5 turns and four turns in males. Buccal cavity with a small dorsal tooth and two pairs of minute ventrosublateral teeth. Spicules paired and slightly ventrally bent, 34 – 46 μm long, gubernaculum with double apophyses and complex crura ridge dorsally with large thorn and lateral protuberances; four large well sclerotized tubiform precloacal supplements; and two short weekly developed tubiform supplements with similar structure between SP1 and cloacal opening. Females with vagina flanked by a pre- and post-vulvar body pore. *Paracanthonchus gynodiporata* sp. n. appears morphologically similar to *P. perspicuus* Kito, 1981 by the presence of a small dorsal tooth, overall spicule shape and gubernaculum structure with a crura ridge with dorsal thorn. However, *P. gynodiporata* sp. n. can be distinguished from *P. perspicuus* by the smaller body length (779 – 1120 μm vs 1269 – 1287 μm), presence of ventrosublateral teeth vs absence in the latter, the presence of only one instead of two crura dorsal thorns, and the presence of two weakly developed precloacal supplements instead of one weakly developed precloacal supplement near the cloacal opening in *P. perspicuus*. Finally, the presence of the pre- and post-advulvar body pores observed in *P. gynodiporata* sp. n. has not been reported in any other species in the literature.

### Description

#### Male (*holotype*)

Body largely cylindrical, slightly narrowing in anterior neck region but more pronounced in conical tail. Punctated cuticular ornamentation, consisting of transverse rows of dots, forming the tip of inner cuticular struts; at the level of the lateral field punctation slightly larger though hardly differentiated in the neck region. Eight longitudinal rows of body pores, at mid body the largest pores bordering the lateral field. Somatic setae arranged in four sublateral longitudinal rows; setae longest (6 μm) and most numerous in neck region. Head region anteriorly rounded and truncated; lip region with six separate lips. Anterior sensilla arranged in two crowns: an anterior crown of six inner labial papillae and an outer crown of six external labial setae (4.5 μm) and four slightly longer cephalic setae (3.7 μm); both types of setae bipartite with open tip. Amphidial fovea spiral (four turns), ventrally wound and surrounded by punctation. No ocelli present. Buccal cavity with cheilostome reinforced by 12 cheilorhabdia and wide cup-shaped; pharyngostome short funnel-shaped with a small well developed dorsal tooth and two pairs of minute ventrosublateral teeth. Pharynx largely cylindrical, just posterior mid-way surrounded by the nerve ring. Outlet of a pair of posterior ventrosublateral pharyngeal glands at the level of the nerve ring; outlet of dorsal gland far anteriorly. Cardia surrounded by intestinal cells and apparently with associated glands; intestine usually with diatoms visible in its lumen. Secretory-excretory pore at short distance from anterior end (26 μm); short outlet sclerotized, swollen anterior ampulla; ventral gland at level of anterior intestine. Tail conical with three well developed caudal glands and nucleus, anteriorly extending along rectum. Spinneret well developed.

Male reproductive system diorchic, anterior testis outstretched on the right side of the intestine, posterior one reflexed and lying on the left side of the intestine; sperm cells small globular (1.5 μm); vas deferens surrounded by muscular sheath and showing differentiation in granulation; spicules paired, slightly ventrally bent, strongly sclerotized with capitulum narrower than blade; blade about equally wide but tapered distally. Gubernaculum, strongly sclerotized and complex structure, composed proximally of a pair of (slightly twisted) apophyses with narrower tip, and crura (wider distal part) embracing retracted spicules, dorsal wall of the crura provided with dentate ridge with one larger thorn and laterally pointed protuberance visible in ventral view. Four oblique anteriorly orientated large, well sclerotized mid-ventral tubiform precloacal supplements; the posteriormost one (SP1) at about 30 μm from cloacal opening; each supplement with a central sensillar canal surrounded by a cuticular wall. In between SP1 and cloacal opening, two short, weakly developed tubiform supplements with similar structure (Fig. 7).

#### Females

General appearance (body shape, cuticular ornamentation), digestive and secretory-excretory system as in male. Head region similar but smaller (but slightly narrower i.e. about 24% of corresponding body diameter in females and 36% in males), fovea with 3.5 turns. Reproductive system didelphic-amphidelphic with antidromously reflexed ovaries, anterior ovary right of the intestine, posterior on the opposite side; uteri with up to three developed oocytes observed in both uteri together; well-developed muscles at level of ovejector. Vagina, rather short, surrounded by vaginal constrictor muscles; vulva at mid-body and flanked by a pre- and post-advulvar body pore. No sperm observed.

#### Remarks

The most important characters to distinguish species in the genus *Paracanthonchus* are the number of teeth in the buccal cavity (Fig. 8) and the number of precloacal supplements [44]. The presence of ventrosublateral teeth is quite variable and was not mentioned in the species descriptions before the 1950’s; it is not clear if teeth have been overlooked or not. The number of precloacal supplements is an easier character to observe and supposedly more reliable. However, the presence and number of minute precloacal supplements near the cloacal opening is an object of discussion.

## Discussion

Paracanthonchus gynodiporata *sp. n. associated with seaweeds shows little population genetic structuring across large geographical distances.*

Our *F*_*ST*_ values show little overall population genetic structure (*F*_*ST*_ = 0.05388). This result was remarkable considering that nematodes in general lack planktonic larvae and dispersal is likely to be limited [14]. Previous population genetic studies showed that nematodes associated with seaweeds have moderate to very large population genetic structuring as observed in a species of *Halomonhystera disjunta* species complex in the region of the Westerschelde estuary (GD3, *Φ*_*ST*_ = 0.11 – 0.13, *p* < 0.001) [9], in species of *Litoditis marina* species complex in the North Sea along the Belgian and Dutch coast (Pm I, *Φ*_*ST*_ = 0.22, *p* < 0.001), from the Bay of Biscay to the Baltic Sea (Pm II, *Φ*_*ST*_ =0.37, *p* < 0.001), and at transatlantic distances (Pm III, *Φ*_*ST*_ = 0.19, *p* < 0.001) [13]. Large population genetic structuring is expected for those two species complexes, because they have very short generation times and high reproductive output [10, 13]. However, very large genetic structuring has also been observed for other seaweed-nematode species, with presumably long generation times and low reproductive output such as for species of the *Thoracostoma trachygaster* species complex (Clade II, *Φ*_*ST*_ = 0.28, *p* < 0.001) [15].

The northern most population, Flecheiras, was differentiated from Muriú (RN) and Cupe. The latter two populations had significant negative neutrality tests and fitted the recent expansion model, suggesting that the differentiation with Flecheiras may be caused by a rapid expansion of the latter two populations. In addition, Flecheiras was the closest population to the border between the northeastern Brazil province and the Amazon province [45, 46]. The proximity of this location to the Amazon river may affect the genetic diversity of the northernmost population, but this remains very speculative. All four locations were dominated by the same nematode haplotype (C8) and showed very similar haplotype networks, with a small number of rare haplotypes and only very few mutations between them. This suggests that all four populations are evolutionary quite young. No geographical structuring was present in the overall network, and together with the weak genetic structuring observed in AMOVA, this suggests that these populations have been well connected. Alternatively, balancing selection may be responsible for the high dominance of the C8 haplotype and the generally low diversity in these populations. Generally, mitochondrial DNA is considered a neutral marker, but it has already been demonstrated that it may be under selection [47]. This would require that selection for the C8 haplotype happened independently in each of the four populations and that not enough time has passed to accumulate new mutations. A second alternative may be the lack of mutation-drift equilibrium, which can also lead to low overall *F*_*st*_ values despite a lack of ongoing gene flow [3, 48]. Especially for low-dispersal species with high effective population sizes, such as marine nematodes, time to reach a mutation-drift equilibrium may take thousands of generations, which may not be achieved in habitats with strong colonization-extinction dynamics such as macro-algal beds.

Although data on the age of seaweed beds in the northeast of Brazil is extremely limited, seaweed beds have a close relationship with coral reefs because many seaweed species need hard substrate to attach and develop [49]. Reef ecosystems in the northeastern coast of Brazil are estimated to have originated around 7 Myr ago, i.e. between the late Miocene and early Pliocene [50], and have been under the influence of sea-level fluctuations during the Pleistocene [51]. However, to what extent those sea-level fluctuations affected the genetic connectivity among populations of marine small metazoans along the northeastern coast of Brazil remains unclear. About 2.7 Myr ago, the Central America Seaway was still open, allowing the Pacific Upper Ocean water to flow towards the Atlantic causing the North Brazil current to flow SE, which is the opposite direction observed today [52]. In this way, the sea current along the northeastern coast of Brazil was continuous and allowed passive dispersal from north to south via rafting on algae. This could explain the presence of similar haplotypes in all populations observed today. The populations of Muriú (RN) beach showed the highest *F*_*ST*_ value in the pairwise comparison with Flecheiras (CE) (*F*_*ST*_ = 0.13125; *p* < 0.001). However, the *F*_*ST*_ values appear to decrease with distance and become insignificant. Such chaotic patterns are not uncommon in marine environments, and adding local environmental data might shed light to understand this apparent chaos [53].

The two main currents at the Brazilian northeastern coast did not appear to constitute a strong physical barrier for *P. gynodiporata* sp. n. as observed by the weak genetic structure between those two regions (*F*_*ST*_ = 0.05204). Our sampling area covered only one biogeographical province, the Northeastern Brazil province. Provinces are classified upon a hierarchical system based on taxonomic configurations, influenced by evolutionary history, patterns of dispersal, and isolation [45]. The lack of large population genetic structure among the *P. gynodiporata* sp. n. agrees well with the above mentioned biogeographical province. In all, our data point to a very low genetic differentiation across a large geographic area suggesting that *P. gynodiporata* sp. n. has performed long-distance dispersal during some time along its evolutionary history. Since the studied species has not been found in the sediment so far [32], drifting seaweeds are known to be used as a dispersal mechanism for diverse marine organisms [26, 54] including nematodes [12]. However this kind of dispersal is limited by the direction of the carrying current and does not fully explain the lack of large genetic structure between locations under the influence of opposite currents in our study. The diverging force of the water currents in this area has to be overcome if mutation-drift equilibrium is present and ongoing gene flow is the major homogenizing force. Nematodes are able to colonize hard artificial substrata [44, 55], for example turtle shells [56], and might thus hitchhike on sea turtles when they forage between seaweed beds [57]. *Paracanthonchus* is a frequent genus found associated with turtles [58] and could possibly feed on epibiont diatoms growing on the turtle shell [59]. Personal recent observations in an *Acanthonchus* nematode species associated with the sea turtle *Eretmochelys imbricata* have shown the presence of an identical mitochondrial DNA haplotype in two beaches more than 900 km apart. Whether the amount of nematodes using this particular dispersal mechanism would be sufficient to establish a population in the new patch remains unclear, as priority effects may hamper the establishment of newly arriving individuals [11].

### Historical exploitation of the natural seaweed bed does not affect the haplotype frequencies of associated nematode populations

Colonization dynamics can strongly impact the mitochondrial haplotype diversity over time [11, 60]. Yet, no variation was observed in genetic composition in Cupe (PE) between years. Moreover, the beach with the highest genetic diversity was the one where historical exploitation of the natural seaweed bed was prominent (Flecheiras (CE)). Commercial seaweed exploitation has gradually disappeared since the 1970s in Brazil and has been replaced from 2002 onwards by seaweed cultivation. It has been argued that seaweed cultivation may increase biological diversity, attracting marine life by creating a harbour where marine species can find shelter and food [61]. In Flecheiras, the fishermen stopped the seaweed extraction from the natural bed and started seaweed cultivation in 2003 which persists until today [23]. Yet, all four populations presented a very similar haplotype diversity and no evidence of founder effects, bottlenecks or genetic drift in *P. gynodiporata* sp. n. was found. Considering that seaweeds were harvested monthly during the peak production period in the 1980’s in the northeastern coast of Brazil [23], the seaweed beds in those regions could also be considered as an ephemeral substrate with a dynamic recolonization rate. It seems the effect of seaweed harvesting on the nematode population is limited: if the population was affected at all, it was able to fully regain its genetic diversity in the 11 years after the harvesting stopped. The source population to re-establish genetic diversity probably came from other seaweed beds since *P. gynodiporata* sp. n. has not been observed in the sediment in the studied locations nor in four other locations along the Brazilian coast (Pirambu (CE), Icapuí (CE), São Sebastião (SP) and Ubatuba (SC)) [32]. Also, historical seaweed exploitation did not lead to genetic changes in nematode haplotype frequencies. This may be caused by the presence of large population sizes, or by substantial gene flow to prevent population genetic structuring even over very large distances (≈1080 km).

### Nematodes can show considerable phenotypic variation among populations potentially biasing species description

There are no synapomorphies at subfamily and genus level within the family Cyatholaimidae [62]. The subfamily Paracanthonchinae shows variation in e.g. lateral differentiation (present or absent) and precloacal supplements (rarely absent). Likewise, within the subfamily, the genus *Paracanthonchus* shares the presence of tubular precloacal supplements with *Acanthonchus,* differing from the latter by the presence of lateral differentiation in cuticular punctation, but no synapomorphy is observed [29]. At species level within *Paracanthonchus*, the difficulty in interpretation of the stomatal armature, especially with respect to the presence and number of ventrosublateral teeth, the number of precloacal supplements, the structure of the gubernaculum as well as the interspecific overlap of morphometric features such as body length and spicule length, complicate species differentiation based on light microscopic observations. This also hampers comparison with older descriptions, in which some features were overlooked or misinterpreted [63]. In Brazil, there are three described species, *P. batidus* [64], *P. digitatus* [64] and *P. cochlearis* [64], which can be distinguished from the new species by the number of precloacal supplements (5 in *P. batidus*; 4 in *P. digitatus*; 4 + 2 *P. gynodiporata* n. sp.) and the number of turns of the amphidial fovea (6 *P. cochlearis*; *P. gynodiporata* n. sp. 4). Substantial overlap exists for other characters (e.g. body length). One possible example of phenotype misinterpretation concerning diagnostic characters is the poorly developed precloacal supplement near the cloaca. In *P. perspicuus*, which is very similar to *P. gynodiporata* sp. n., Kito [65] claimed that the poorly developed supplement is a single structure with a single opening, while two tubular-like structures are illustrated. However, in the new species we observed the presence of two poorly developed tubular precloacal supplements, each with its own opening. It is unclear, however, whether the presence or absence and number of those very small precloacal supplements in other *Paracanthonchus* species are a result of misinterpretation or not. The combination of the following characters could be used to distinguish between species from the genus *Paracanthonchus*: 1) number of precloacal supplements including the poorly developed tubular supplement near the cloaca, 2) the mouth armature including the number of ventrosublateral teeth, 3) the ornamentation of the gubernaculum (e.g. ridges, thorn like protuberances) and 4) the number of loops in the amphidial fovea. Body length, body width and pharynx length should be given less weight.

Interestingly, the observed phenotype variability is not accompanied by genotypic variability in *P. gynodiporata* sp. n. A threshold between intra and interspecific genetic distances for the COI gene in marine nematodes has been set at 4.8% p-distance [66, 67]. In our work, the highest difference between haplotypes was 1.5%. A combination of morphological characters differentiated the four studied *P. gynodiporata* sp. n. populations and non-overlapping morphometric characters such as buccal cavity width, distance of amphidial fovea from anterior end, and cephalic setae length were able to differentiate populations. It has been demonstrated that body and tail length can substantially vary within a single species progeny [68] and even the presence or absence of teeth in a single species [69] can be affected by environmental variables (e.g. food source), but it has only rarely been documented from field collected specimens. In contrast to the substantial phenotypic variability observed, a maximum of 6 haplotypes per location per year was observed, with 17 haplotypes in total over more than 1000 km. Surprisingly, the nuclear sequences were identical for all individuals of the two studied locations Cupe (PE) and P. Verde (AL). The opposite pattern (high genetic variation and no morphological variation) is well documented in a wide range of species [70], including marine nematodes [14, 55, 60, 71, 72]. Due to the limited number, small size and the high risk of losing individuals of *P. gynodiporata* n. sp. during voucher procedure, we have not used the same individuals for DNA sequencing and morphometry. However, because of the low haplotype richness (maximum of 6 haplotypes per location) and the high dominance of a single haplotype, at times representing more than 80% (Muriú (RN)) of the haplotype frequencies, it is very likely that the individuals used for the morphometry are from the same or similar haplotypes. Because of initial differences in number of adults among the four populations, we have added a number of juveniles to increase the balance of our design for the molecular analysis. However, because 1) only one species of the genus *Paracanthonchus* occurred associated with seaweeds in our samples in the four studied locations, and 2) very few overall mutations were present among their sequences (maximum of six mutations out of 396 bp for COI, and identical nuclear D3D3 and 18S sequences between two beaches), it is highly unlikely that the added juveniles belonged to a different species.

Many morphological traits are encoded by multiple genes, and it is possible that other regions of the genome could show more variation than the three genes we have studied. Moreover, environmental factors can play a role in nematode phenotypic variation without similar levels of genetic differentiation as a result of epigenetic mechanisms (e.g. methylation of CpG sites - cytosine followed by a guanine with one phosphate in between) [73]. For instance, reduced activity of the Hsp90 (a heat shock protein) caused morphological variations in an isogenic *Drosophila melanogaster* strain [74], showing that epigenetic modification can be expressed in the organism’s morphology. Such changes can be heritable and may imply that in natural populations, mutation is not the only source of heritable variation [73], and epigenetic changes are also an important mechanism underlying microevolutionary processes.

Clearly, an integrative approach using independent data sources can lead to scientifically valid species delineation. Some morphological characters of nematodes are difficult to observe. Similarly, the genus *Paracanthonchus* presents a wide variation in mouth structure [29]. The pairs of ventrosublateral teeth can be strongly reduced and barely visible in lateral view, and consequently they could have easily been overlooked using light microscopy in previous publications [64, 75]. Ideally, morphometric data from different populations, as provided in this study, should be included to give an idea of the morphological variability of a species. However, it is comprehensible that this is not always possible if sampling requires intensive logistic effort (e.g. deep sea). Our data provides another clear-cut example of the need to combine multiple approaches (morphology and DNA sequences) to describe and determine species boundaries.

## Conclusion

Nematodes associated with seaweeds can show low genetic structuring over large distances (>1000 km), suggesting dispersal capacity of nematodes can be high throughout the evolutionary history of the species. There is no evidence that historical seaweed exploitation has affected genetic diversity or haplotype frequencies of epiphytic marine nematodes. Morphometric variation in natural populations can be substantial, showing interspecific overlap, and one should combine at least molecular and morphological data in an integrative way to establish species boundaries and describe diversity.
